# Radiation-induced heart disease: a review of classification, mechanism and prevention

**DOI:** 10.7150/ijbs.35460

**Published:** 2019-08-08

**Authors:** Heru Wang, Jinlong Wei, Qingshuang Zheng, Lingbin Meng, Ying Xin, Xia Yin, Xin Jiang

**Affiliations:** 1 Department of Radiation Oncology, The First Hospital of Jilin University, Changchun, 130021, China; 2 Department of Cardiology, The First Hospital of Jilin University, Changchun, 130021, China; 3 Department of Internal Medicine, Florida Hospital, Orlando, FL 32804,USA; 4 Key Laboratory of Pathobiology, Ministry of Education, Jilin University, Changchun 130021, China

**Keywords:** Radiation-induced heart disease, inflammation, oxidative stress, statins, ACE inhibitors.

## Abstract

With the increasing incidence of thoracic tumors, radiation therapy (RT) has become an important component of comprehensive treatment. RT improves survival in many cancers, but it involves some inevitable complications. Radiation-induced heart disease (RIHD) is one of the most serious complications. RIHD comprises a spectrum of heart disease including cardiomyopathy, pericarditis, coronary artery disease, valvular heart disease and conduction system abnormalities. There are numerous clinical manifestations of RIHD, such as chest pain, palpitation, and dyspnea, even without obvious symptoms. Based on previous studies, the pathogenesis of RIHD is related to the production and effects of various cytokines caused by endothelial injury, inflammatory response, and oxidative stress (OS). Therefore, it is of great importance for clinicians to identify the mechanism and propose interventions for the prevention of RIHD.

## Introduction

With an increase in the incidence of tumors, radiation therapy (RT) has become an important treatment method 1. RT improves survival of patients with tumor, but it also involves some inevitable complications of radiation. Radiation-induced heart disease (RIHD) is one of the most serious complications. Previous studies demonstrated that the heart is well resistant to radiation, and the symptoms of RIHD often require a long incubation period to manifest, so the RIHD has not attracted much attention 2. With the increasing number and the prolonged survival of patients, researchers have gradually found that the RIHD offset some benefits and many patients succumbed to ischemic heart disease 3-5. RIHD is now receiving increasing attention from clinicians and patients.

RIHD comprises a spectrum of heart disease including pericarditis, cardiomyopathy, coronary artery disease, vavular heart disease, and cardiac conduction abnormality 6. Up to now, the total morbidity of RIHD has not been completely censused in the throng of patients with thoracic tumors. However, because of the high 5-year survival rate of breast cancer and Hodgkin's lymphoma patients, the current research on the incidence of RIHD mainly focuses on patients with the above two types of cancer. Because the latency of each disease and the follow-up time in each study are different, the results of studies are quite different. The incidence of each kind of heart disease varies greatly among breast cancer patients, ranging from 0.5% to 37% 7-9. In lymphoma patients, the incidence of RIHD is higher than that of breast cancer patients, reaching 49.5-54.6%, and the incidence of various heart diseases ranges from 11-31%^10^-^12^. For other thoracic tumors, true morbidity of RIHD was underestimated because of its short survival rate and short follow-up time. And a number of large clinical studies have confirmed that radiation therapy increases the risk of heart disease-related mortality^12^, ^13^. Although morbidity of RIHD has been reduced by optimized treatment plan and techniques with radiation, but recent studies have shown that modern technology does not eliminate the risk of RIHD^9^, ^14^.

At present, it is thought that RIHD is a result of various mechanisms interacting with each other through multiple complex pathways. However, endothelial injury, oxidative stress (OS) and inflammation, endoplasmic reticulum and mitochondrial damage are considered to be the main reasons 1. With the development of research, microRNAs(miRNAs) have gradually become a new focus of research on the pathogenesis of RIHD 15. Many researchers believe that miRNAs regulate the production of various cytokines, which in turn play an important role in the development of late cardiac injury related with radiation. There is no clear treatment program to effectively eradicate the onset and subsequent development of RIHD. However, reducing heart exposed range and doses have become a recognized primary prevention for RIHD. When the heart has been irradiated secondary prevention is crucial.

In this review, we summarize the common classification and important mechanisms that cause RIHD as well as some treatments.

## 1. The common types of RIHD

RIHD following chest RT has been recognized fact and demonstrated in numerous clinical trials. The choice of treatment and tumor location has significant influence on RIHD^16^, ^17^. At the present stage, chemotherapy is a risk factor for RIHD, anthracycline had evident effects on RIHD^18^. Studies have clearly indicated that receiving chemotherapy or radiotherapy alone is more facility to suffer ischaemic heart disease, and chemoradiotherapy increases risk of arrhythmia and heart failure^16^. The dose is linearly associated with the morbidity of RIHD, tumor location makes it extremely variable. The left anterior myocardium, pulmonary valve and atrioventricular are strongly impacted by the left radiation^17^. In addition to the above factors, age, smoking, diabetes, hypertension, dyslipidaemias, obesity etc are also applied to the common types and morbidity of RIHD^14^.

### 1.1 Pericarditis

The clinical process of pericarditis can be divided into 4 stages including acute and chronic pericarditis, fibrinous pericarditis , and the final evolution, constrictive pericarditis^19^.The most frequent manifestation of acute stage is exudative pericarditis 2. Its occurrence is mainly related to damage of capillary endothelial cells and lymphatic stenosis or occlusion^20^, ^21^. Before the optimization of RT techniquset and scheme, about 80% of patients receiving RT suffered acute pericarditis^22^, ^23^. Many of patients with pericardial effusion present with hemodynamic abnormalities, but in most cases it is self-limited. The presence of a clear, benign pericardial effusion in acute phase may predispose patients to chronic pericarditis. But only 20% of patients developed symptomatic constrictive pericarditis.^24^. The morbidity is closely related to the radiation dose received by the heart. When the radiation dose is increased by 10 Gy, the morbidity increases five times^25^. Although the incidence of pericarditis has decreased to 6-10% with the optimization of radiotherapy protective techniques and programs, studies have shown that the risk of pericarditis among breast cancer survivors is still increasing^8^, ^26^.

### 1.2 Cardiomyopathy

The clinical symptoms of myocardial injury caused by RT are quite late, mainly manifested as myocardial fibrosis^27^. Studies demonstrated that the incubation period of myocardial injury can be as long as more than ten years, and by the time of diagnosis most patients have previously experienced irreversible damage^28^. Most radiation-induced myocardial injury has no clinical symptoms, so the clinically diagnostic rate is low, only about 10%^26^. The most common echocardiographic abnormalities are regional wall motion abnormality (usually inferior wall), mild left ventricular hypertrophy and diastolic dysfunction, which can manifest as severe congestive cardiac insufficiency^29^. Myocardial injury is common in patients who have received anthracycline chemotherapy or high dose of radiation (>60 Gy). Patients who have received high dose of radiotherapy are prone to restrictive myocardial injury, and who have received chemoradiotherapy are prone to diastolic myocardial injury^30^.

### 1.3 Coronary artery disease (CAD)

The injury of coronary artery induced by radiation is consistent with coronary atherosclerosis due to additional factors. The initial trigger was still endothelial cell injury the infiltration of monocytes into the intima, inducing low-density lipoprotein deposition and the formation of fatty streaks 31, 32. RT as an external influence factor can induce the microvascular injury and accelerate the onset of CAD with high-risk patients 33. RT induces vascular endothelial dysfunction, which ultimately results in clinical cardiovascular events that occur many years later after completion of RT. However, with the extension of patients' survival and the attention to RIHD, many clinical studies are dedicated to this field. Clinical studies have demonstrated that the incidence of CAD in patients is up to 85%, it closely related to radiation dose, location, time, and other factors 3, 8. As for high-dose radiotherapy, a study conducted by Netherlands Cancer Institute found that compared with the general population, the cumulative incidence of CAD over 25 years was 34.5% and its risk increased 3-5 times 11.

### 1.4 Valvular heart disease

Myocardial ischemia and hypoxia caused by myocardial fibrosis and coronary diseases are the basic causes of valve function injury. The incubation period of RT induced valvular heart disease (VHD) is much longer than the aforementioned types of RIHD. Therefore, VHD lesions are very rare clinically. A previous study of Hodgkin's lymphoma survivors showed a cumulative VHD incidence of 10% in 13 years increasing to 20% in 30 years. This suggested that previous history of RT increased the likelihood of VHD in these patients to a large extent 11. The incidence of VHD is closely related to the doses and anthracycline. Dose decrease resulted the gradual decline in the accumulated VHD incidence over 30 years from 12.4% at >40 Gy to 3% at <30 Gy 34. The earliest changes in the general pathology of VHD seem to include valvular contraction and associated reflux within the first 10 years after RT, with preferential involvement of the mitral and aortic valves. The progression of valvular fibrosis thickening and calcification occurs much later, with stenosis often occurring 20 years after RT 35. Mitral regurgitation and aortic regurgitation are the most common defects. When stenosis occurs, aortic regurgitation is frequently involved 36.

### 1.5 Conduction system abnormality

Conduction system abnormality caused by RT usually manifests as atrioventricular block, pathological sinus node syndrome, QTc prolongation, supraventricular arrhythmia and ventricular tachycardia^37^. The incidence of conduction system abnormality is about 5%, often occurs within 2 months after the end of RT, and 70% of ECG abnormalities can return to normal after half year of RT, but the incidence rate was still increased compared with that before the treatment^33^, ^38^. This shows that the effect of RT on the heart is partly reversible, but it will still cause some damage to heart.

## 2. Pathogenesis of RIHD

**Figure 1:**
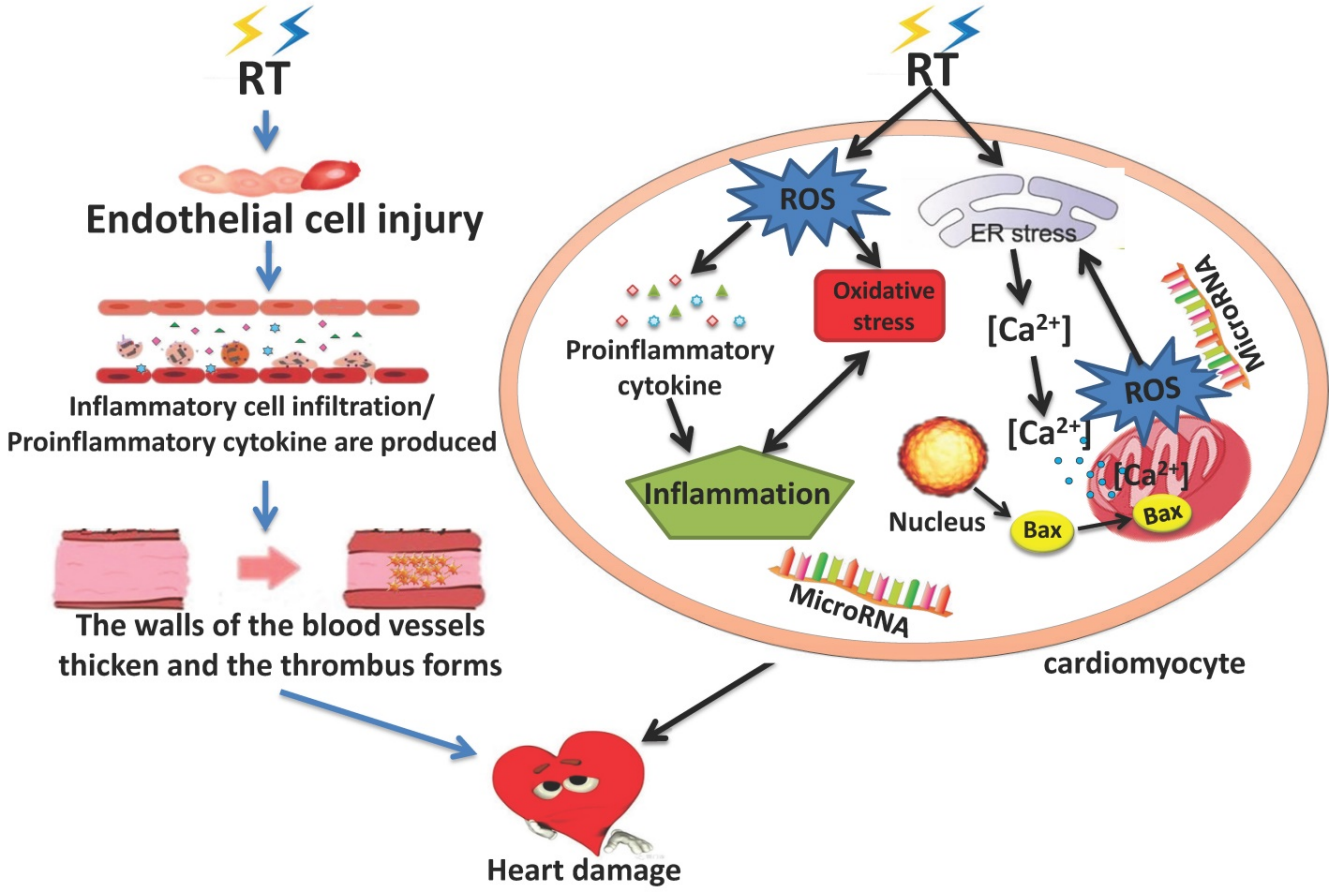
The Pathogenesis of RIHD

Although the effect of radiation on the heart has been clear in pre-clinical trials, the underlying mechanism of RIHD gradual progression from no clinical manifestations in the early stage to chronic heart disease in the later stage is not fully understood. There are many cytokines involved in the process and the regulation and control mechanisms are affected by various factors. These factors interact with each other so that the mechanism of RIHD is very complex. At present, it is believed that RIHD is associated with endothelial cell injury, inflammatory reaction, OS, mitochondria and endoplasmic reticulum injury, various cytokines, calcium overload, and micro-RNAs 21, 27, 39. It is accepted that the early damage of RT is mostly caused by acute and chronic inflammatory changes, and the late toxicity is partly caused by OS and inflammation together. These changes can lead to heart disease^40^. Understanding the biological mechanism of RIHD is very important to clarify the pathogenesis of related diseases, and it will also be an important step to evaluate the feasible therapeutic targets. (Figure 1)

### 2.1 The endothelial cell injury and inflammation

Radiation-induced endothelial cell injury is deemed to be the primary and fundamental cause of myocardial injury^41^-^44^. RT can influence cardiac capillary endothelial cells, leading to their proliferation, injury, swelling and degeneration, and significantly reduce the number of capillaries. Although endothelial cells can regenerate, capillary network damage is irreversible 45, this may reduce the blood supply of myocardium. Radiation exposure of heart not only induces endothelial cell damage and the decrease of capillaries, but also changes coagulation function and platelet activity. The deposition and release of von Willebrand factor (vWF) in endothelial cells increased after radiation exposure of heart. The changes of vWF expression eventually lead to increasing platelet adhesion and thrombosis in capillaries^46^, ^47^. Animal studies have also shown that the inflammatory thrombotic plaque emerged in the blood vessels after the rat heart exposured to high doses of radiation^48^.Thrombosis and decreased cardiac blood supply together lead to myocardial ischemia^49^.

Both *in vivo and vitro* experiments showed that, in addition to the increased expression of vWF, the expression of e-selectin, p-selectin, intercellular cell adhesion molecule (ICAM), plaet-endothelial cell adhesion molecule-1(PECAM1) and other pro-inflammatory adhesion factors also increased a few hours after the endothelial cells exposure to radiation, mediating the infiltration of inflammatory cells into tissues and promoting the acute inflammation^50^-^54^. The regulation of irradiation-induced pro-inflammatory adhesion factors may be the key to early endothelial response. In addition to the increased expression of these adhesion factors, inflammatory mediators such as tumor necrosis factor-α, interleukin (IL-6, IL-8 and IL-10) also appeared in the myocardium to participate in the formation of acute inflammation.

Experiments have shown that IL-8 can not only mediate inflammatory responses, but also induce apoptosis. Then platelet-derived growth factor (PDGF), transforming growth factor Beta(TGF-β), nuclear factor kappa B (NF-κB), and connective tissue growth factor (CTGF) are released, leading to the chronic inflammation. 55-58.

The above inflammatory factors can not only mediate the production of inflammation, but also promote the proliferation of endothelial cells and fibroblasts, the increase of collagen deposition can cause the thickening of vessel walls and the stenosis of lumen^41^, ^42^. This may exacerbate the lack of blood flow to the myocardium due to the reduced capillary network. The heart is the main oxygen consuming organ in the human body, decreased blood supply can give rise to myocardial hypoxia which will aggravated the myocardial injury. Myocardial ischemia and hypoxia, inflammatory responses, collagen deposition, and proliferation of endothelial cells and fibroblasts lead to tissue remodeling, cardiac fibrosis, and atherosclerosis, and these changes are the primary endpoints of RIHD^49^, ^59^.

### 2.2 Oxidative stress

In normal cell, reactive oxygen species (ROS) are important mediators of cellular processes such as immune response, cell signal transduction, microbial defense, differentiation, cell adhesion and apoptosis^60^, therefore, the production of ROS is beneficial to cells under physiological conditions^61^. When ROS are produced in large quantities, their activity can be eliminated by reduction of intracellular antioxidants, including glutathione, to remove excess free radicals 62, 63. However, glutathione and other antioxidants are also consumed during their activity, and the cell's ability to maintain redox balance is ultimately impaired.When the amount of endogenous and/or exogenous ROS exceeds the scavenging capacity of antioxidants, ROS begins to dominate and cause damage to cardiac myocytes. Some scholars believe that ROS - mediated OS is an important cause of atherosclerosis, hypertension and congestive heart failure^64^, ^65^. Like other heart diseases, OS also plays an important role in RIHD^36^. 80% of tissues and cells are composed of water, and most of the radiation damage (X-ray, gamma rays, rapid electrons) after exposure to radiation is caused by the generation of ROS and reactive nitrogen species (RNS) caused by the radiation decomposition of water, which is an important source of normal tissue damage after ionizing radiation 66. DNA damage is likely to occur when intracellular antioxidants cannot adequately remove ROS. It has been reported that DNA damage has many forms, which can significantly change the structure of DNA and eventually lead to cell cycle arrest, apoptosis, mutation and other effects^67^, ^68^. The DNA damage repair (DDR) pathway, mediated by multiple functional proteins in cells, is an important mechanism to repair DNA damage and ensure the integrity of the genome^69^. P53 gene is one of the main effectors of DDR signaling pathway^70^. Same as P53, Bcl-2 gene family also changes after radiation, leading to increased apoptosis^71^. In addition to DNA damage, ROS can also lead to peroxidation of lipids and proteins and activate multiple signaling pathways^72^.

ROS can not only directly damage the intracellular macromolecular structure, but also altered the expression of multiple proteomes in the cytoplasm, activation of pro-inflammatory factors in connection with ROS^73^. As a second messenger signal in cells, ROS participates in and regulates signaling pathways, including mitogen-activated protein kinases(MAPK) and NF-κB, and promote the occurrence of inflammation^74^, ^75^. NF-κB regulates DNA transcription and protein complexes engage in various cellular stress responses, and may be a key regulator of the link between OS and inflammatory^76^. ROS acts as a second messenger to activate NF-κB and induce the production of inflammatory cytokines. Therefore, proinflammatory cytokines and chemokines are believed to be closely related to the occurrence of OS, while OS enhanced inflammation in turn drives the progression of disease, leading to a vicious cycle 77.However, how OS and inflammation interact to promote RIHD remains unclear.

### 2.3 Apoptosis(endoplasmic reticulum and mitochondria signaling pathway)

Cell apoptosis and necrosis occur in various types of cells in the heart after exposure to radiation, among which mitochondrial dysfunction and irreversible damage are the key links of cell apoptosis and necrosis, and the occurrence of mitochondrial dysfunction is closely related to endoplasmic reticulum(ER)stress. Mitochondria are organelles that account for an important proportion of the total volume of cardiac myocytes, and mitochondria carry extranuclear DNA, so they are important targets for radiation-induced cell damage^78^. Mitochondrial permeability transition (MPT) and loss of mitochondrial membrane potential are important mechanisms of mitochondrial dysfunction and are involved in the pathogenesis of a variety of cardiovascular diseases^79^. Multiple stimuli, such as calcium ions flowing into mitochondria, inorganic phosphates, reactive oxygen species and other oxidants, can also induce MPT^80^. After cardiac myocytes are irradiated, the stimulated ER releases calcium ions from the calcium pool of the ER into the cytoplasm. This process will cause mitochondrial calcium overload and lead to its membrane swelling and release of apoptotic factors from it. Moreover, severe MPT can lead to mitochondrial membrane depolarization and the decoupling of oxidative phosphorylation, which is closely related to the opening of mitochondrial permeability transition pore(mPTP)^39^, ^81^. Bax is one of the important pro-apoptotic proteins in the Bcl-2 family 82. It has been reported that exposure to RT leads to increased expression and activation of Bax, leading to its translocation and insertion into the mitochondrial outer membrane 83, 84. This accelerated the opening of mitochondrial voltage - dependent anion channels. MPT and the insertion and ectopia increase of Bax improve the permeability of mitochondrial membrane and reduce the mitochondrial membrane potential together. This prolongs and enhances calcium-induced mitochondrial membrane swelling, leading to apoptosis^71^.

Excessive ROS production by mitochondria in human cells was observed immediately after irradiation^64^. A large amount of ROS can cause lipid peroxidation and protein damage of ER. Then the ER produces a small amount of ROS and releases it into the cytoplasm. These reactions can reduce mitochondrial membrane potential, inhibit respiratory chain, and accelerate the generation of peroxidation^39^, ^85^, ^86^. The increased permeability of mitochondrial membrane leads to a cascade reaction, which produces a large amount of ROS. ROS further promotes the release of calcium from the calcium pool of the ER, leading to the overload of calcium in mitochondria, thus increasing the generation of ROS. This is consistent with the results of cell experiments. Oqura et al. found that once the acute increase of ROS subside, the subsequent generation of ROS will be observed^87^. Kobashigawa et al. also pointed out that ROS levels produced by mitochondria continued to increase one week after radiation exposure^88^. A vicious cycle of ROS produced by mitochondria and Ca^2+^ release caused by ER may lead to long-term toxicity induced by radiation, which eventually leads to cell cycle arrest^68^. This leads to apoptosis and premature aging. In addition, mitochondrial damage can also cause bystander effect in neighboring mitochondria, which amplifies radiation effect and leads to further cell damage 39, 89.

### 2.4 Micro-RNA

With the development of research, several studies have indicated that micro-RNAs (miRNAs) play an important role in the occurrence and progression of RIHD 90-92. Alterations in miRNA expression may occur following exposure to several OS-inducing factors including ionizing radiation 90. Many studies confirmed that miRNAs are implicated in the pathological processes connected with cardiac radiation damage, OS, inflammation, endothelial dysfunction, hypertrophy, and fibrosis resulting in heart failure 91, 92. Recently, miRNAs have been found to be involved in the regulation of radiation-induced DNA damage and the induction of premature aging^93^. MiRNA-21 has the function of promoting cell proliferation and anti-apoptosis 94. Csilla et al. found that the expression of miRNA-21 in the myocardium was significantly increased after radiation and this change was more obvious in the left ventricle than in the right ventricle 95. MiRNA-1 expression was altered in many cardiovascular diseases and down-regulated in irradiated animal models, consistent with changes in cardiac hypertrophy and heart failure 91. Furthermore, changes in miRNA-34a expression are also related to heart injury. A study showed that miRNA-34a expression was up-regulated after the heart was exposed to radiation 96.

In addition to the above mi-RNAs, miRNA-29, miRNA-199b, miRNA-221, miRNA-222, and miRNA-15 are also believed to be associated with the occurrence of heart disease 97-99. However, it is not clear whether they are related to RIHD. The role of miRNAs in RIHD is a relatively new research topic, which has significant therapeutic potential in clinic. Much work has been done on miRNAs as important regulators of the cardiovascular system, and the understanding of their role in RIHD is currently limited. Further studies are needed to clarify the mechanisms underlying the regulation of RIHD by miRNAs.

## 3. Drugs therapy of RIHD

There is currently no effective treatment for RIHD, in particular for the prevention of over-exposure. Improvements in radiotherapy regimens to decrease exposure to normal healthy tissue near tumor cells are considered to be primary prevention 18. As early as in the 1980s, technologies for reducing cardiac radiation dose have been applied in clinical practice, such as deep inspiratory breath hold (DIBH), three-dimensional conformal radiotherapy, intensity modulated radiation therapy, and volumetric arc therapy etc., which greatly reduce the radiation dose and volume received by the heart during radiotherapy^100^-^102^. However, the heart inevitably receives radiation doses during RT. It may be impossible for cardiovascular tissues to be fully protected, therefore secondary prevention (follow-up visits for patients and drug treatment) is crucial 103. Studies suggest that RIHD can be prevented by using some drugs including statins, ACE inhibitors, and antioxidants 104-106. (Table 1)

### 3.1 Statins

Statins are 3-hydroxy-3-methylglutaryl coenzyme A reductase inhibitors that are used in clinical practice to reduce cholesterol and lipoprotein density. Recent studies have shown that statins, in addition to lowering cholesterol, can reduce OS and activate adenosine 5'-monophosphate-activated protein kinase (AMPK) to achieve anti-inflammatory effects. Consequently, stains can protect the heart by inhibiting inflammatory reactions and OS 107, 108. Researchers of previous studies found that pravastatin not only inhibited the early inflammation of the lungs caused by bleomycin, but also reduced the expression of transforming growth factor (TGF)-β1, CTGF, RhoA, and cyclin D1^109^. This means that in addition to lowering blood lipids, statins not only inhibit inflammation and oxidative stress, but also reduce the production of tissue fibrosis. The protective effect of statins on the heart against radiation damage was consistent with the above results.

In the acute response period after radiation exposure to normal tissues, lovastatin can inhibit the activation of transcription factor NF-κB and the expression of inflammatory cell adhesion molecules, thereby inhibiting the acute inflammatory response. For the latter chronic toxicity phase, lovastatin can inhibit the mRNA expression of fibrotropic factor CTGF which induced by RT, and the formation of tissue fibrosis may be alleviated by this change^110^, ^111^. Pravastatin can also inhibit tissue fibrosis caused by radiation, but its inhibitory ability is weaker than that of lovastatin^112^. Zang et al. further confirmed through experiments that atorvastatin can reduce radiation-induced myocardial fibrosis by inhibiting multiple inflammatory responses and OS pathway activation 104. Doi et al. showed that pravastatin could also protect tissue damage caused by radiation by reducing DNA double-strand breakage in normal tissue cells 113. However, it has been reported that atorvastatin can improve the repair of oxidative DNA damage in vascular smooth muscle cells (VSMCs) without affecting the initial level of DNA damage^114^. Therefore, the protective effect of statins on RT-induced myocardial injury is probably related to the repair of DNA damage. A few studies have been conducted on their application in radiation-induced myocardial injury 95, 115, but statins have the potential to be effective protectors of myocardial radiation. Therefore, it is important to clarify the mechanism of action for the discovery of RIHD protection targets.

### 3.2. Angiotensin-converting enzyme inhibitors (ACEIs)

The renin-angiotensin-aldosterone system (RAAS) is known to play an important role in cardiac remodeling. ACEIs in the RAAS system not only inhibits the production of ROS to reduce myocardial injury caused by OS and inflammation, but also increases the production of NO to protect vascular cells by reducing the negative effects on the bradykinin system 116. ACEIs are usually used to treat hypertension or congestive heart failure. Studies have indicated that ACEIs may ameliorate radiation-induced toxicity in different organs, including the heart 117, central nervous system 118, and lungs 119.

Interestingly, ACEI drugs can reduce myocardial perivascular fibrosis and myocardial cell apoptosis through anti-inflammation and reducing oxygen free radicals after simultaneous exposure of the heart and lungs, thereby inhibiting myocardial fibrosis and decreased cardiac diastolic function 105. Rats treated with captopril shortly after the lung was exposed to radiation demonstrated dramatically increased survival and improved vasoreactivity, as well as decreased perivascular fibrosis and inflammatory cell infiltration 119. Furthermore, rats treated with captopril exhibited reduced diastolic dysfunction and perivascular necrosis in the left ventricle following radiation 105. There are many differences between research and clinical radiation therapy, but clinical trials have also indicated that ACEI drugs can reduce the incidence of pneumonia induced by radiotherapy 120. Although these data are interesting, prospective studies evaluating the efficacy of ACEIs in patients undergoing radiation have not been reported. In order to further clarify whether ACEI drugs can be a protective agent to reduce RIHD, a large number of clinical research patients would be required.

### 3.3. Anti-inflammation and Anti-OS compounds

Inflammation and OS play an important role in the development of RIHD and they interact with each other in various ways. Colchicine inhibits the inflammatory response by inhibiting microtubule polymerization and can reduce platelet aggregation, protecting the heart through its anti-inflammatory and anti-coagulant properties 106. Some Chinese herbal extracts have been shown to inhibit the inflammatory response induced by radiation and the formation of myocardial fibrosis 121, 122.

As described above, ROS and RNS are released in large quantities after irradiation, which promotes an acute inflammatory response and subsequent OS. A pre-clinical trial indicated that rats exposed to 7 Gy gamma radiation and injected with caffeic acid phenethyl ester (CAPE), had an suppressed acute immune system and inflammatory response, as well as induced antioxidant properties, thereby alleviating the myocardial injury caused by radiation^123^. Tocomin SupraBio (TSB) enriched with tocotrienols can retain stability of the membrane potential and confront radiation-induced alterations in succinate driven mitochondrial respiration in the rat model of local heart exposed to irradiation. TSB also significantly improved the redox state and maintain the ratio of pro-apoptotic protein Bax and Bcl-2 through regulating the ratio of GSH and GSSH in left ventricular tissues. Therefore TSB can relieve radiation-induced mitochondrial changes and achieve anti-OS and anti-apoptosis in hearts. However, this drug cannot effectively inhibit the generation of radiation-induced myocardial fibrosis in the later stages, so whether it can be applied in clinical practice remains unknown^124^. A combination of antioxidant pentoxifylline and α-tocopherol inhibited myocardial fibrosis in irradiated rats by inhibiting expression of intracellular TGF-β and CTGF. In addition, pentoxifylline also changes endothelial function and prevents downregulation of endothelial cell surface thrombomodulin to defends endothelial function^125^. It's worth noting that nuclear factor (erythroid-derived 2)-like 2 (Nrf2) is a transcription factor which encodes many antioxidants and anti-electrophile enzymes. The activation of p38MAPK/Nrf2 signaling expression and the activation of downstream pathways may significantly suppress the degree of OS, reduce myocardial injury, and protect cardiac function^126^. In addition, there are many drugs such as melatonin and amifostine that are thought to reduce radiation-induced heart toxicity through anti-inflammatory and anti-OS, but the underlying mechanisms require further study 127, 128.

### 3.4. Others

It is well known that TGF-β is not only involved in inflammation and OS, but also induce collagen deposition and play an important role in the formation of myocardial fibrosis. Irradiated rats were given xaliproden (an orally active non-peptide agonist) to increase circulating TGF-β1 levels by 300% which significantly induced the expression of TGF-β1 and TGF-β1 target genes in the heart tissue. Similarly, in the same RIHD model, induction of TGF-β1 augmented radiation-induced changes in cardiac function and myocardial fibrosis 129. IPW-5371, as a TGF-βR1 inhibitor, was reported to reduce collagen deposition in the heart and lungs and significantly improve the cardiopulmonary function of mice after irradiation 130. These results further demonstrate the direct involvement of TGF-β1 in radiation-induced adverse remodeling and damage in the heart.

In addition, a recent animal trial has also shown that rhNRG-1β can reduce the damage to myocardial nuclei caused by radiation, maintain mitochondrial homeostasis, improve energy metabolism of myocardial cells, and alleviate the reduction of cardiac function and cardiac structural changes 131. Furthermore, GSTA4-4 can eliminate Nrf2 activator 4-HNE and reduce the activation of antioxidant stress pathway. The cardiac function and capillary density of GSTA4-4 KO mice were improved compared with WT mice and the expression of Nrf2 transcription factor was up-regulated after receive local radiation. Therefore, GSTA4-4 inhibitors and recombinant Nrf2 activators have also become research hotspots for anti-OS drugs which can reduce cardiac radiation toxicity 117. However, the mechanisms of these drugs are complex and indistinct. Further clinical trials and studies must be conducted before these drugs are actually used in clinical settings.

## 4. Conclusion

Survivors receiving chest radiotherapy are at an increased risk of RIHD. RIHD represents a spectrum of cardiac pathology including CAD, Cardiomyopathy, pericardial disease, arrhythmias, and valvular abnormalities. Although pre-clinical animal and cell models have been used to study the potential pathophysiological mechanisms of RIHD, the exact mechanisms of the various RIHD pathogenesis are not entirely understood. We have reviewed several common pathways involved in the development of RIHD including endothelial injury, inflammation and OS, and endoplasmic reticulum and mitochondrial dysfunction. The development of therapeutic targets to prevent microvascular damage, inflammation, and late fibrosis will hinge on our increased understanding of RIHD. The use of certain drugs can be quite helpful in reducing radiation-induced heart damage. However, these drugs may be not the most accurate treatment for RIHD and need to be developed for specific disease progression.

## Figures and Tables

**Table 1:** Summary of studies investigating the effect of different drug classes on RHID

Drug class	Drug	Target	Observations	References
Statins	Atorvastatin	Atorvastatin reduced the expression of TGF-β1, Smad3/P-Smad3, ROCK I and p-Akt	Atorvastatin Ameliorate radiation-induced cardiac fibrosis in Sprague-Dawley rats.	Zhang et al.^70^
ACEIs	Captopril	Captopril inhibits the the renin-angiotensin system and scavenges free radical	Captopril improves breathing rate and cardiopulmonary density/structure	van der Veen et al.^71^
Anti-inflammation and Anti-OS compounds	Tocotrienol-rich mix	Tocotrienol-rich mix protects mitochondrial dysfunction	Tocotrienol-rich mix relieves mitochondrial changes and achieves anti-OS and anti-apoptosis in hearts.	Sridharan et al.^89^
	Pentoxifylline + α-tocopherol	Pentoxifylline and α-tocopherol inhibits expression of intracellular TGF-β and CTGF and defends endothelial function	Pentoxifylline and α-tocopherol inhibit myocardial fibrosis	Boerma et al.^90^
	caffeic acid phenethyl ester (CAPE)	No description	CAPE suppresses acute immune system, inflammatory response and induces antioxidant properties	Mansour et al.^44^
	L-carnitine	L-carnitine administration activated p38MAPK/Nrf2 signaling	L-carnitine attenuates cardiac function loss	Fan et al.^91^
	Melatonin	Melatonin scavenges free radical	Melatonin prevents vasculitis and decreases fibrosis and necrosis	Gurses et al.^92^
	Amifostine	No description	Amifostine prevents vascular damage and vasculitis	Kruse et al.^93^
TGF-β1 inhibitors	IPW-5371	IPW-5371 antagonizes TGF-βR1	IPW-5371 reduces collagen deposition in the heart and lungs and significantly improve the cardiopulmonary function of mice after irradiation	Rabender et al.^95^
Recombinant human neuregulin-1	rhNRG-1β	rhNRG-1β activats ErbB2-ERK-SIRT1 signaling transduction	rhNRG-1β reduces myocardial damage and protects heart function	Gu et al.^96^
